# Nanoparticles for Bioapplications: Study of the Cytotoxicity of Water Dispersible CdSe(S) and CdSe(S)/ZnO Quantum Dots

**DOI:** 10.3390/nano9030465

**Published:** 2019-03-20

**Authors:** Fatemeh Mirnajafizadeh, Deborah Ramsey, Shelli McAlpine, Fan Wang, John Arron Stride

**Affiliations:** 1 School of Chemistry, University of New South Wales, Sydney, NSW 2052, Australia; f.mirnajafizadeh@unsw.edu.au (F.M.); d.ramsey@unsw.edu.au (D.R.); s.mcalpine@unsw.edu.au (S.M.); 2 School of Mathematical and Physical Sciences, University of Technology Sydney, Ultimo, Sydney, NSW 2007, Australia; Fan.Wang@uts.edu.au

**Keywords:** bioapplications of QDs, HCT-116, WS1, core/shell QDs, water dispersive QDs, aqueous synthesis, in vitro cytotoxicity of QDs

## Abstract

Semiconductor nanocrystals or quantum dots (QDs) have unique optical and physical properties that make them potential imaging tools in biological and medical applications. However, concerns over the aqueous dispersivity, toxicity to cells, and stability in biological environments may limit the use of QDs in such applications. Here, we report an investigation into the cytotoxicity of aqueously dispersed CdSe(S) and CdSe(S)/ZnO core/shell QDs in the presence of human colorectal carcinoma cells (HCT-116) and a human skin fibroblast cell line (WS1). The cytotoxicity of the precursor solutions used in the synthesis of the CdSe(S) QDs was also determined in the presence of HCT-116 cells. CdSe(S) QDs were found to have a low toxicity at concentrations up to 100 µg/mL, with a decreased cell viability at higher concentrations, indicating a highly dose-dependent response. Meanwhile, CdSe(S)/ZnO core/shell QDs exhibited lower toxicity than uncoated QDs at higher concentrations. Confocal microscopy images of HCT-116 cells after incubation with CdSe(S) and CdSe(S)/ZnO QDs showed that the cells were stable in aqueous concentrations of 100 µg of QDs per mL, with no sign of cell necrosis, confirming the cytotoxicity data.

## 1. Introduction

Semiconductor nanocrystals or quantum dots (QDs) have received a great deal of attention over the last decade due to their unique optical and physical properties. This has led to them being classified as a powerful new class of bio-imaging tools [1,2,3,4,5,6,7,8,9,10]. Despite the desirability of using QDs as intense fluorescent nanoscale markers in biological applications, cytotoxicity is a serious constraint that limits potential uses [11,12,13]. QDs can be considered as being potentially toxic due to both their nanoscale size and the presence of heavy metals [12,13]. Nanoscale particles can have a greater toxicity than bulk counterparts, with the small size enabling greater penetration into cells than corresponding bulk chemical materials [14]. In addition, the generation of free radicals, such as reactive oxygenated species (ROS), upon cell exposure to nanomaterials is a significant cause of nanoparticle cytotoxicity [14,15,16,17]. Interactions of QDs with cell mitochondria and nuclei can result in the disruption of cell function, inhibition of cell proliferation, and decreased cell viability due to ROS production, ultimately resulting in cell death, mutation, or induced immunotoxicity [15,18,19,20,21]. The long-term toxicity of QDs has also been attributed to bioaccumulation in organs, leading to organ damage and chronic illnesses [22]. The high ratio of reactive surface area relative to the bulk generally increases the chemical activity of nanoparticles, which can result in an accumulation of nanoparticles in tissues and associated organ toxicity [23], particularly those having high blood flow, such as spleen, kidneys, liver, and lungs, along with the blood circulation system itself [24]. Therefore, QDs have largely been classified as potentially poisonous to both human and animal cells, and as such, cytotoxicity assays of QDs are an essential requirement before applying QDs to cellular environments.

There are various methods to determine the toxicity of QDs in both in vivo and in vitro studies. In vivo studies may involve introducing QDs to microorganisms [25] or the use of animal models [26,27,28,29,30], whereas in vitro toxicity features the treatment of various cell types with QDs in order to investigate the cytotoxicity of nanocrystals in mammalian cell lines [31,32,33,34,35,36,37,38,39,40,41,42,43]. A common feature in all reports of cadmium-based QDs, including CdTe, CdSe, and CdS, is that Cd is a primary source of toxicity. Accordingly, core/shell QDs such as CdTe/CdS, CdTe/CdS/ZnS, and CdSe/ZnS have been shown to exhibit less toxicity than core QDs alone [13,37]. However, the cytotoxicity of QDs depends on a number of parameters, including the surface modifications of the QDs, cell type, cellular morphology, cell growth, and the interaction of QDs with cell membranes [36,42].

There are extensive reports detailing cytotoxicity assays of QDs synthesized in organic media [28,35,36,37,38,39,40,41,42,43,44,45], but few papers have reported in detail the cytotoxicity of QDs directly obtained from aqueous solution [13,46,47,48,49]. Bhatia and co-workers investigated the in-vitro cytotoxicity of organically synthesized CdSe QDs on liver cells and showed that the release of free cadmium ions from the QDs resulted in cell death [36]. Meanwhile, Zhu et al. studied both the in vivo and in vitro cytotoxicity of aqueous CdSe and CdSe/CdS QDs and reported that the toxicity of QDs depends on both target cells and physicochemical properties of the QDs [48]. Plank et al. investigated the cytotoxicity of CdSe and CdSe/ZnS QDs obtained in organic solvents, demonstrating that QD cytotoxicity is related to both particle size and the surface covering of functional groups, such as amines and carboxylic acids used to disperse them in water [38]. Fan et al. investigated the cytotoxicity of a wide range of QDs (CdTe, CdTe/CdS, and CdTe/CdS/ZnS) synthesized in aqueous reactions, concluding that free cadmium ions are a major source of toxicity in CdTe-based QDs, also reporting that CdTe/CdS/ZnS QDs were slightly less toxic than CdTe in their experiments, due to effective protection of the ZnS shell [13]. The present work reports on the cytotoxicity of CdSe(S) and CdSe(S)/ZnO QDs, synthesized in wholly aqueous reactions, to human colorectal carcinoma cells (HCT-116) and human skin fibroblast cells (WS1). To reduce the cytotoxicity of QDs, two strategies were used, including synthesis of QDs using an aqueous synthetic method and also protection of the core against oxidation with formation of core/shell QDs. The water dispersible QDs were synthesized in a modified literature method [50] and the cytotoxicity of both the QDs and the precursor solutions were determined after incubation of HCT-116 cells with the QDs over a wide range of concentrations (25 to 500 µg/mL). Confocal images of HCT-116 cells after treatment with QDs were recorded and cytotoxicity of QDs to WS1 cells was also studied to explore the action of QDs towards normal, resilient cell types.

## 2. Experimental Methods

### 2.1. Materials

The 3-mercaptopropionic acid (MPA), CdCl_2_·2.5H_2_O, Zn(CH_3_COO)_2_·2H_2_O, NaBH_4_, Se (powder), NaOH, bisbenzimide (Hoechst 33342), 4′,6-diamidino-2-phenylindole (DAPI), and Rhodamine 6G were all obtained from Sigma-Aldrich Pty. Ltd (Sydney, Australia). Human colorectal carcinoma cells (HCT-116) and human skin fibroblast cells (WS1) were obtained from ATCC Inc. (Manassas, VA, USA). Dulbecco’s modified eagle medium (DMEM), fetal bovine serum (FBS), penicillin, streptomycin, L-glutamine, and nonessential amino acids were received from Invitrogen Inc., (Carlsbad, CA, USA). All reagents were used as supplied, without additional purification. Ultra-pure water was used in all syntheses.

### 2.2. Methods

#### 2.2.1. Synthesis of QDs

Water dispersible CdSe(S) and CdSe(S)/ZnO QDs were synthesized and characterized using optimized experimental parameters in a modified literature method [50], as detailed in Supplementary Materials Data S1.

#### 2.2.2. Characterization of QDs

UV-vis absorption spectra were measured with a Varian Cary UV spectrometer (Agilent Inc., Santa Clara, CA, USA). Photoluminescence spectra were measured on a Carry Eclipse fluorescence spectrometer (Agilent Inc., Santa Clara, CA, USA) using an excitation wavelength of 350 nm. X-ray powder diffraction patterns (PXRD) were recorded on an X’pert PRO Multi-purpose X-ray diffraction system (MPD system) with a Cu Kα source (*λ* = 0.154056 nm) manufactured by Malvern Panalytical Pty. Ltd (Chipping Norton, NSW, Australia). X-ray photoelectron spectroscopy (XPS) was performed using an Escalab 250Xi spectrometer (Azo Materials Analysis Inc., Madison, WI, USA) using a mono-chromated Al Kα X-ray source (*hν* = 1486.6 eV) operated at 10 kV and 10 mA. High resolution transmission electron micrographs (HRTEM) were obtained using a Philips CM200 instrument (Philips FEI Inc., Hillsboro, OR, USA). Dynamic light scattering (DLS) was used to estimate the average hydrodynamic diameters of CdSe(S) QDs using a Malvern Instruments Zetasizer Nano ZS instrument (Malvern Panalytical Pty. Ltd., Chipping Norton, NSW, Australia), equipped with a 4 mV He-Ne laser operating at *λ* = 633 nm with a high quantum efficiency avalanche photodiode detector and an ALV/LSE-5003 multiple tau digital correlation function; the data obtained were analyzed using Malvern software.

#### 2.2.3. Preparation of Aqueous Solutions of QDs

A total of twenty different solutions of CdSe(S) and CdSe(S)/ZnO QDs, Cd-MPA (Sample 1, Supplementary Materials Data S1), and Cd-Se-MPA (Sample 2, Supplementary Materials Data S1) were prepared by diluting the aqueous solutions with ultra-pure water to achieve concentrations of 25, 50, 100, 250, and 500 µg/mL. Each sample was used in cytotoxicity assays without further purification. In order to remove excess cadmium ions and capping agent from the aqueous solution, a Slide-A-Lyzer dialysis cassette (ThermoFisher Pty. ltd., VIC, Australia) was used in the QD stock solution and the cytotoxicity of dialyzed CdSe(S) QDs in the presence of HCT-116 cell line was investigated in five samples at concentrations of 25, 50, 100, 250, and 500 µg/mL.

#### 2.2.4. Cytotoxicity Assays

Cell cultures of human HCT-116 and WS1 cell lines were obtained according to the standard protocol [51], as detailed in Supplementary Materials Data S2. The cells were then separately seeded in two 96-well plates (3000 cells/well) and allowed to adhere to the dish for 24 h in a humidified incubator. As control experiments, cell media (3000 cell/well) without QD treatment was used under identical experimental conditions, according to other cytotoxicity assays reported in literature [52]. Finally, 10 µL of each aqueous solution was added to the plates. The plates were incubated in the presence of the QDs for 72 h at 37 °C with 5% CO_2_, after which the samples were analyzed to determine the cell proliferation using a Cell Counting Kit-8 assay (Sigma-Aldrish Pty. Ltd., Sydney, Australia).

#### 2.2.5. Confocal Microscopy Studies

The first two samples of either fixed or live HCT-116 cells-QDs were prepared (Supplementary Materials Data S3). Images of the cells in the presence of QDs were recorded under Leica TCS SP5 CW STED (Leica Microsystems Inc., Wetzlar, Germany) and Zeiss LSM 780 confocal microscopes (Zeiss Inc., Feldbach Switzerland), respectively. The live cells were stained by adding a Hoechst solution (5 µg/mL) 10 min before recording images, whilst the fixed cells were stained using DAPI. In addition, the emission profiles of QDs in both cell media and aqueous solution were recorded using a Zeiss LSM 780 confocal microscope. The images of QDs in cell media were also recorded.

## 3. Results and Discussion

### 3.1. Synthesis of QDs

The method of synthesizing QDs can play an important role in the overall toxicity, with literature reports indicating that QDs synthesized in organic solvents tend to be more toxic than those synthesized by aqueous pathways [53,54,55,56]. However, the toxicity of Cd-based QDs has largely been attributed to the free Cd ions existing in equilibrium with the QDs in solution [13,36,57]. Coating the QD cores with appropriate shell materials may prevent the core from oxidation, thereby reducing the number of free Cd ions released [48,58,59,60,61,62]. In this work, an aqueous hydrothermal method reported by Aldeek and co-workers [50] was used to synthesize CdSe(S) and CdSe(S)/ZnO QDs using optimized experimental parameters, leading to the formation of highly crystalline QDs.

**Figure 1:**
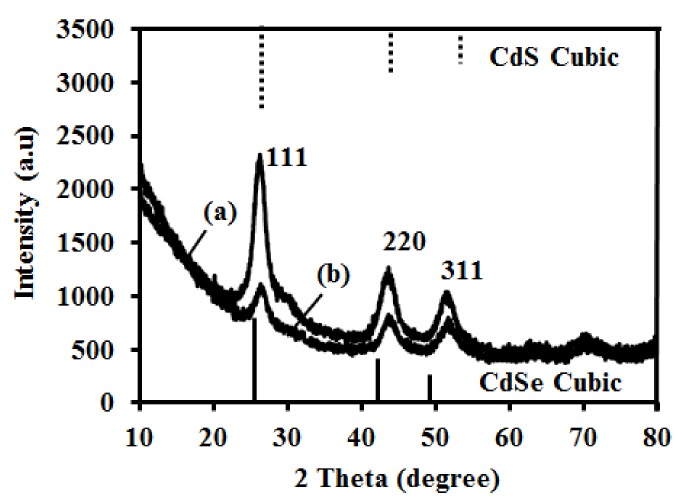
PXRD of as- synthesized QDs: (**a**) CdSe QDs and (**b**) CdSe(S)/ZnO QDs.

PXRD patterns of the synthesized QDs showed that both CdSe(S) and CdSe(S)/ZnO QD cores had the cubic zinc blende structure (Figure 1), according to the standard CdSe cubic pattern [63]. The diffraction peaks of the QDs were found to be consistent with standard patterns of the cubic phases of CdSe [63] and CdS [64] and are characteristic of alloyed CdSe-CdS, as shown in Table S1, Supplementary Materials Data S4. As ZnO is amorphous, there was no peak observed in PXRD of CdSe(S)/ZnO QDs that can be attributed to ZnO. The rationalization of ZnO forming an amorphous ZnO shell rather than a crystalline one is that crystalline ZnO is obtained from the hydrolysis of appropriate precursors but only following additional treatments, such as pulsed-laser deposition, high temperature annealing, or thermal cycling [65,66]. In the present experiments, ZnO was obtained from the hydrolysis of zinc acetate at basic pH and without any additional processing, and as such can be considered to be amorphous, in accordance with previous reports [50]. Moreover, the quantum yield (QY) of the core CdSe(S) QDs was estimated to be around 3% compared with the QY of Rhodamine 6G in water (95%), whilst that of the coated CdSe(S)/ZnO QDs was 0.6%, as detailed in Supplementary Materials Data S6. The decrease of QY in CdSe(S)/ZnO QDs is consistent with enhanced nonradiative relaxation due to sequential internal conversion steps between intra-gap defect states in amorphous ZnO. Finally, PXRD data of the CdSe(S)/ZnO QDs showed none of the peaks expected for a ZnO standard crystalline phase [67], indicating the absence of crystalline ZnO in the as-synthesized Cdse(S)/ZnO QDs. In conclusion, the cubic structure of CdSe(S) QDs remained intact after coating with an amorphous ZnO shell, in accord with a previous report [50].

The size of the core was estimated using the Scherrer equation as 3.6 ± 0.1 nm and 3.0 ± 0.1 nm in CdSe(S) QDs and CdSe(S)/ZnO QDs, respectively. This indicates a potential decrease in the size of the QD core during the heating process in the formation of the zinc oxide shell. The decreased particle size of the CdSe(S) QD core after heating using reflux is in accord with our previous studies on CdSe nanoparticles [68]. However, the total size of CdSe(S)/ZnO QDs cannot be estimated by using the Scherrer equation because the ZnO shell is amorphous.

**Figure 2:**
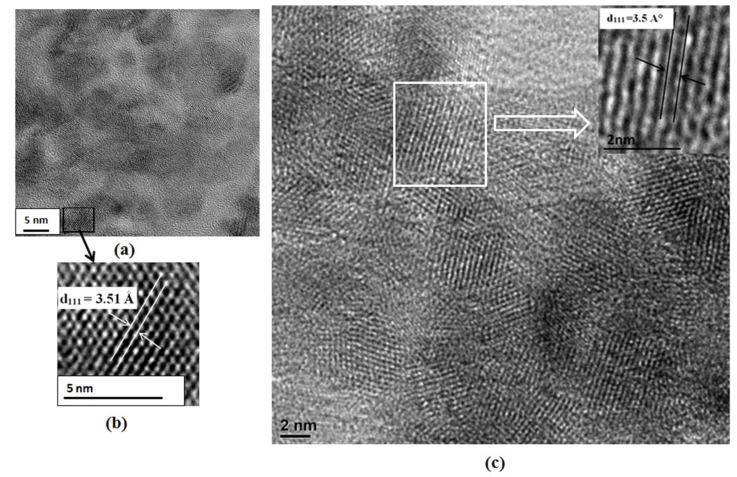
HRTEM images of QDs: (**a**,**b**) CdSe QDs and (**c**) CdSe(S)/ZnO QDs.

HRTEM images of the crystalline NPs (Figure 2) showed evidence of atomic planes with a d-spacing equal to 3.51 ± 0.01 Å in CdSe(S) QDs and 3.5 ± 0.01 Å in CdSe(S)/ZnO QDs. These relate to the (111) planes in cubic CdSe, in accord with the PXRD data, which relate to the (111) planes in standard cubic CdSe [63]. As the obtained QDs were aggregated during the recording of the TEM images, they could not be seen as distinct nanoparticles. In fact, dried samples imaged under TEM were found to be strongly aggregated, largely due to interactions between corresponding groups in the capping agent, MPA. This is in distinct contrast to solubilized samples, where the capping agent assists in aqueous dispersion. The dispersed particle size distribution was determined using DLS and the obtained size distribution histograms showed that the QDs are well dispersed in water (Supplementary Materials Data S5).

Similar to previous reports [50,62], HRTEM did not show the existence of the shell due to the small size of nanoparticles and aggregation. The thickness of the shell was estimated by considering that the total volumes of the CdSe and ZnO components are equal, based upon the fact that the XPS data highlights similar elemental compositions, then from the core particle size of 3.0 nm estimated the shell thickness is simply $\sqrt[{3}]{2r_{c}^{3}}$-$r_{c},$ where *r_c_* = the core radius = 1.5 nm, giving a shell thickness of ~0.4 nm.

**Figure 3:**
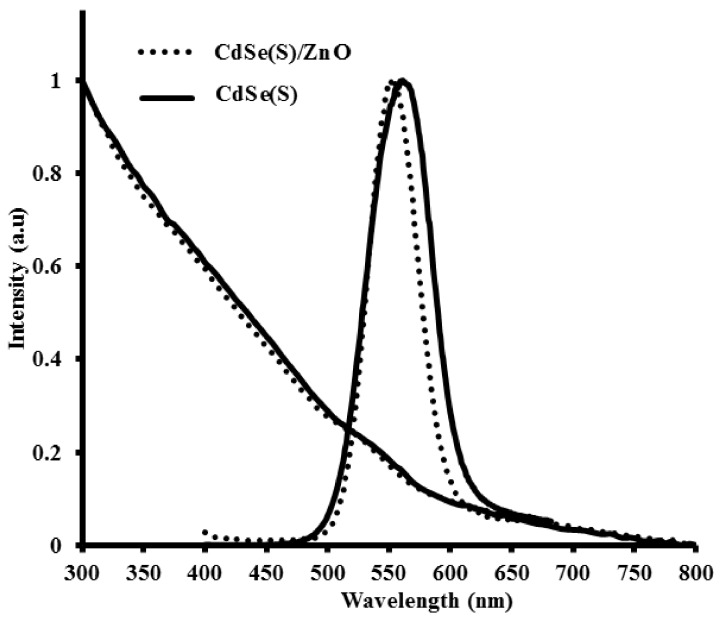
Optical spectra of CdSe(S) and CdSe(S)/ZnO QDs.

As-synthesized CdSe(S) and CdSe(S)/ZnO QDs had strong emissions under UV light (Supplementary Materials Data S7) with narrow emission peaks at 560 and 550 nm for CdSe(S) and CdSe(S)/ZnO QDs, along with broad excitation bands starting at 530 nm for CdSe(S) and 520 nm for CdSe(S)/ZnO QDs, respectively (Figure 3). The wide band absorption and narrow emission of QDs are mainly related to the specific structure of QDs. The structure of QDs is intermediate between bulk semiconductor and atoms. In bulk semiconductors, the atomic orbitals overlap to form continuous energy levels in both the valance band (VB) and conductive band (CB). Electrons then occupy all of the states up to the edge of the VB, whilst the electronic states in the CB are almost empty and only occupied by thermal excitation across the energy gap (*E_g_*). In semiconductor QDs, the energy states are quantized due to quantum confinement effects. In QDs, when a photon has an excitation energy exceeding the semiconductor band gap, the QD may absorb the photon, creating an excited electron in the CB, resulting in a wide-band absorption, usually in the UV-visible region [69]. The excited electron can relax to its ground state by the emission of another photon with energy equal to the band gap, resulting in a narrow and symmetric emission known as photoluminescence (PL) [70,71]. UV-Vis spectra of CdSe(S) and CdSe(S)/ZnO QDs both had long tails, extending into the near-infra-red (NIR) region, along with weak first excitation bands. The reason can be attributed to defect states that energetically lie within the band gap. However, due to the small Stokes shift, these defects do not contribute to the emission pathway.

The optical properties of the QDs are wholly related to the CdSe(S) QD cores, with the ZnO present as an amorphous shell, exhibiting no optical properties in this region, evidenced by the low QY of CdSe(S)/ZnO QDs (0.6%), being only 20% of that of the core CdSe(S) QDs (Supplementary Materials Data S6). As mentioned previously, the low QY of CdSe(S)/ZnO QDs is fully consistent with an amorphous ZnO shell, as the optical properties of the core/shell QDs are predominantly related to that of the CdSe(S) cores, albeit with some loss in QY. It must be noted however that both the CdSe(S) and CdSe(S)/ZnO QDs exhibited relatively low QYs; this has been observed in previous reports, where despite the advantageous nature of aqueous synthetic methods, the resulting QDs suffer from lower QYs [52,72].

The emission wavelength of the CdSe(S) QDs (*λ* = 560 nm) (particle size = 3.6 ± 0.1 nm) was found to be slightly different to that of CdSe QDs (*λ* = 515 nm) (particle size = 2.5 ± 0.5 nm) reported previously [50], consistent with both the larger particle size and modified Cd:MPA molar ratio used. Moreover, the emission wavelengths of CdSe(S)/ZnO QDs (*λ* = 550 nm) were found to shift to lower wavelengths relative to the cores CdSe(S) QDs (*λ* = 560 nm), in agreement with the estimated particle size (3.0 ± 0.1 nm) but in contrast to a previous report [50]. This is primarily due to the sensitivity of QDs on changes in environmental or experimental parameters. For example, Aldeek and co-workers [50] reported that the emission wavelengths of CdSe(S)/ZnO QDs shifted to lower wavelengths compared to uncoated CdSe(S) QDs after 4 min of UV exposure [50], indicating that even small changes in temperature or illumination can lead to shifts in emission and excitation wavelengths of the QDs.

**Figure 4:**
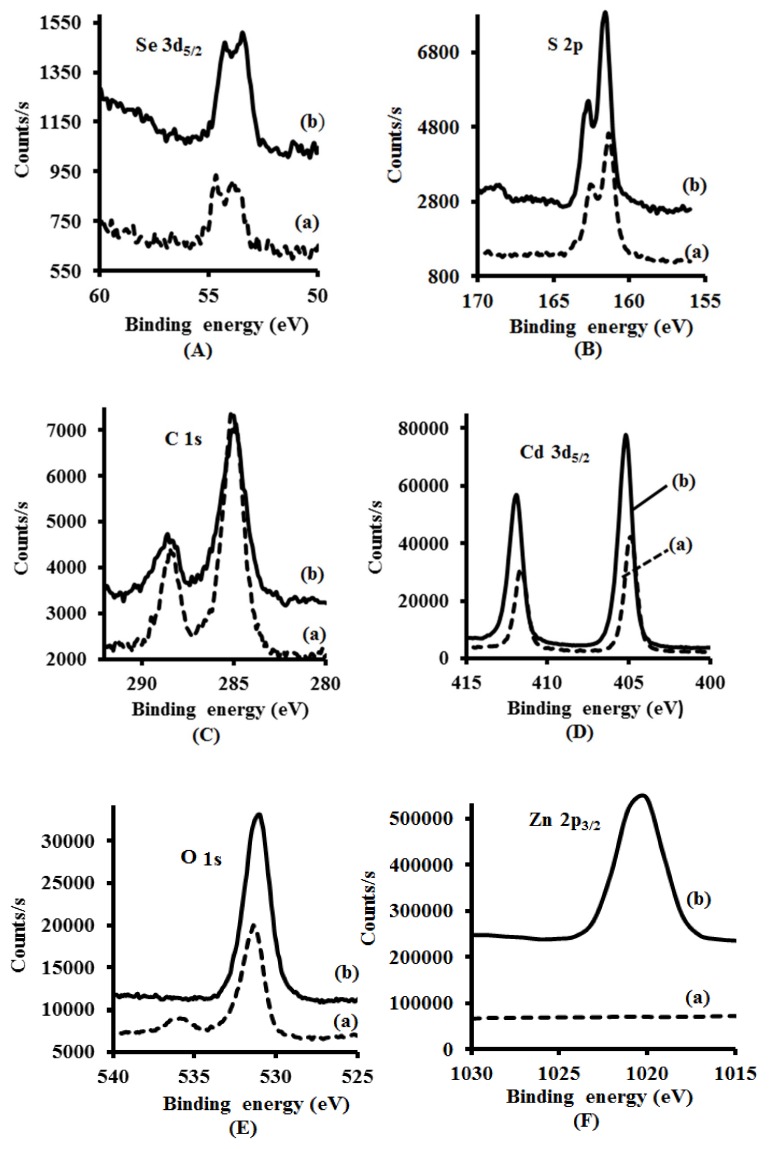
XPS spectra of QDs: (**a**) CdSe(S) and (**b**) CdSe(S)/ZnO QDs: Binding energy of (**A**) Se_3d_, (**B**) S_2p_, (**C**) C_1s_, (**D**) Cd_3d_, (**E**) O_1s_, and (**F**) Zn_2p_.

The XPS spectra of both CdSe(S) and CdSe(S)/ZnO QDs (Figure 4), confirmed the existence of the peaks assigned to Se 3d_5/2_ at 52 eV, Cd 3d_5/2_ at 404 eV, S 2p at 161.8 eV, O 1s at 532, and three C 1s peaks at 284.5, 286.5, and 288.5 eV, indicating that the shell growth does not influence the structure of CdSe(S) QD cores. Meanwhile, XPS data of CdSe(S)/ZnO QDs showed a peak at 1022 eV assigned to Zn 2p_3/2_, as evidence for existence of zinc in the structure of CdSe(S)/ZnO core/shell QDs. The peak characteristics of S 2p at 161.8 eV in the XPS data of the QDs is in agreement with previous reports in the literature, where MPA releases sulfur at high temperature [73,74], resulting in sulfur from the 3-mercaptopropionic acid (MPA) participating in the growth of CdSe(S) particles. Indeed, XPS data of CdSe(S)/ZnO QDs confirmed the existence of both Zn and O in the core/shell CdSe(S)/ZnO QDs, with the observation of a new peak related to Zn 2P_3/2_at 1022 eV (Figure 4F), indicating the formation of a ZnO shell around the CdSe(S) cores; this is consistent with the standard X-ray photo electron spectrum of ZnO [75] and is in accord with previous work [50].

XPS analysis was also used as a quantitative method to determine the elemental compositions of samples. The atomic percentages of as-prepared QDs have been summarized in Table 1.

This confirmed that CdSe(S) QDs were coated with a ZnO shell. The CdSe(S) QDs contained cadmium (13.7%), selenium (0.7%), sulfur (13.1%), oxygen (29.9%), and carbon (42.5%), with the ratio of atomic percentages C:O:S = 3.2:2.3:1, fully consistent with the molecular composition of the capping agent MPA, C_3_O_2_SH_6_. The atomic percentage of Cd was found to be approximately equal to the sum of the atomic percentages of the Se and S contributions, indicating that at the surface of the CdSe QDs, the MPA coordinates by substitution of S at the Se sites. The low intensity of the Se peak suggests a low atomic percentage of Se relative to that of S (1:18.2) and is due to the dominance of surface atoms in the XPS data, essentially MPA and the outer surface of the CdSe(S) QDs. Besides, according to quantitative analysis data (Table 1), the most abundant element in the CdSe(S)/ZnO QD spectrum was O (37.7 atomic %). Each MPA molecule accounts for two O and one S atom. The atomic % of S was found to be 12.3%, and so MPA accounts for 24.6% of the O contribution, leaving 13.1%, which closely matches the 12.0% contribution of Zn. The Cd:(Se + S) ratio of 1:0.8 and Cd:Zn of 1:0.7 are fully consistent with a ZnO shell around the CdSe(S) core. In addition, the peaks of Cd and Se shift with the incorporation of ZnO, highlighting the modified bonding environments at the interfacial atoms. The ratio of core to shell in the as-synthesized CdSe(S)/ZnO core/shell QDs was estimated to be about 1:1. This is based on quantitative elemental analysis; in CdSe(S)/ZnO QDs, the total atomic % of Cd, S, and Se is 29.7%, whilst the total atomic % of oxygen is 37.7%, incorporating both MPA and ZnO. As the MPA formula is C_3_H_6_O_2_S, the ratio of carbon to oxygen is 3 to 2, and so the atomic % of O in the CdSe(S) core and ZnO shell are 13.7% and 24%, respectively. Therefore the total atomic % of core is 29.7%, whilst total atomic % of Zn and O in the ZnO shell is 25.7%. Therefore, the ratio of core to shell is around 1:1.

### 3.2. Cytotoxicity Assays

The responses of HCT-116 and WS1 cell lines upon exposure to the QDs were studied in order to investigate toxicity. Cancer cells, such as HCT-116 cells, have an irregular DNA pattern, making them more sensitive than healthy cells to free heavy metals, including cadmium. In contrast, normal skin cells (WS1) are known to be some of the most resistant cell types to free metal ions. The cytotoxicity of as-synthesized QDs was determined towards both HCT-116 and WS1 cell lines at different concentrations. This cytotoxicity was assessed based upon the measurement of cell viability after incubation in the presence of QDs.

#### 3.2.1. Cytotoxicity of CdSe(S) QDs towards HCT-116 Cells

**Figure 5:**
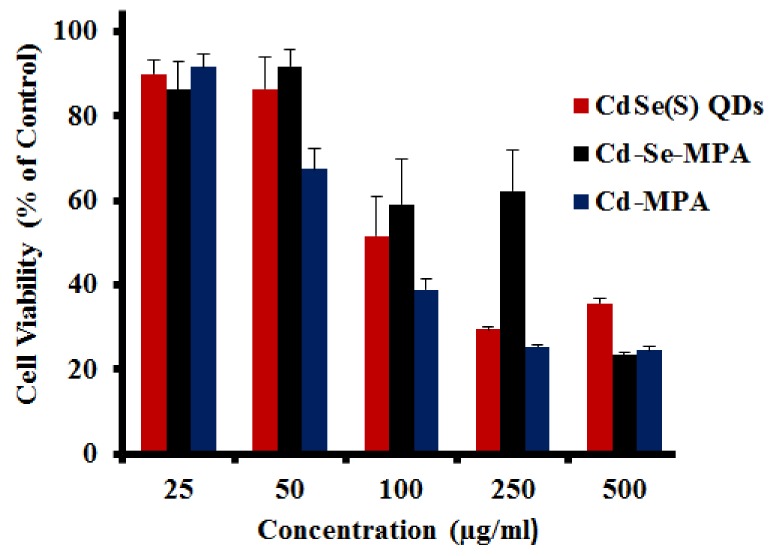
The results of cytotoxicity assays of CdSe(S) QDs, Cd-MPA, and Cd-Se-MPA precursors towards human colorectal carcinoma cells (HCT-116). Error bars indicate standard error of the mean and cell media without treatment with QDs was used as a control.

The lethal concentration corresponding to the death of 50% of cells (LC_50_) was determined as 105 µg/mL for the HCT-116 cell line upon exposure to CdSe(S) QDs. CdSe(S) QDs were found to have a low toxicity at the highest dilutions studied (25 and 50 µg/mL), with 89.9 ± 3.4% and 86.4 ± 7.7% cell viability, respectively, but have a cell viability rate of less than 50% at a concentration of 250 µg/mL and 500 µg/mL, with a dose-dependent cytotoxicity (Figure 5).

#### 3.2.2. Cytotoxicity of Precursor Solutions

The CdSe(S) QDs were synthesized by hydrothermal reactions of two precursor solutions containing Cd-MPA and Cd-Se-MPA. The viability of HCT-116 cells was measured to both of the precursor solutions (Figure 5), which were found to have no significant toxicity at concentrations of 25 and 50 µg/mL, but to be cytotoxic at a concentration 500 µg/mL, similar to that of CdSe(S) QDs. The Cd-MPA solution was found to be more toxic than the Cd-MPA-Se precursor solution. In addition, cells treated with the Cd-MPA precursor solution had a lower viability (67.3 ± 4.9%) at 50 µg/mL than the Cd-Se-MPA precursor (92.0 ± 3.9%), indicating that formation of the Cd-Se-MPA complex decreases the toxicity, presumably due to a decreased number of free cadmium ions.

#### 3.2.3. Cytotoxicity of Dialyzed CdSe(S) QDs

**Figure 6:**
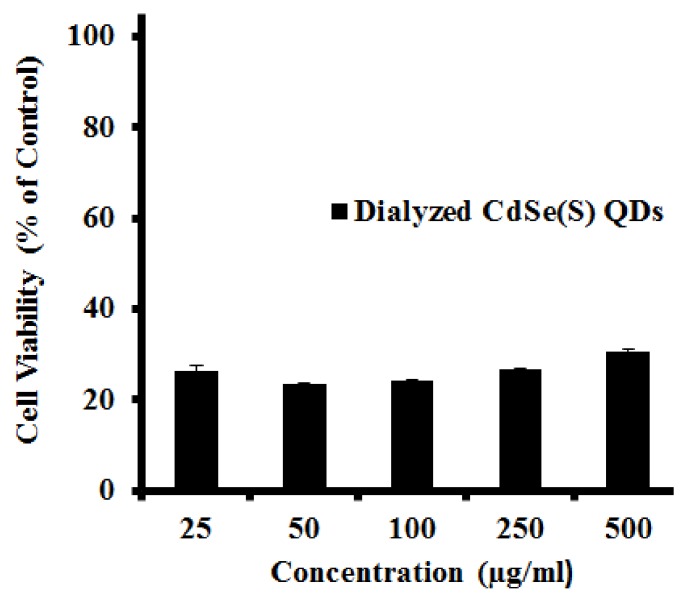
The results of cytotoxicity assays of dialyzed CdSe(S) QDs towards human colorectal carcinoma cells (HCT-116). Error bars indicate standard error of the mean and cell media without treatment with QDs was used as a control.

A dialysis cassette was used to remove excess capping agent and cadmium ions from solution, however the results showed that the CdSe(S) QD solutions were, in fact, more toxic after dialysis than before (Figure 6). This is consistent with a report of CdTe QDs with thiol capping agents, in which a surface cadmium-thiol shell protected the QDs against oxidation, resulting in greater stability of the QDs [76]. In aqueous solution, QDs are in an active equilibrium with excess cations and thiol in solution. During dialysis, excess thiol and cadmium ions are removed from the solution, resulting in the destabilization of the QD surface, thereby leaching more free cadmium ions into the solution, increasing toxicity.

#### 3.2.4. Cytotoxicity of CdSe(S)/ZnO Core/Shell QDs

**Figure 7:**
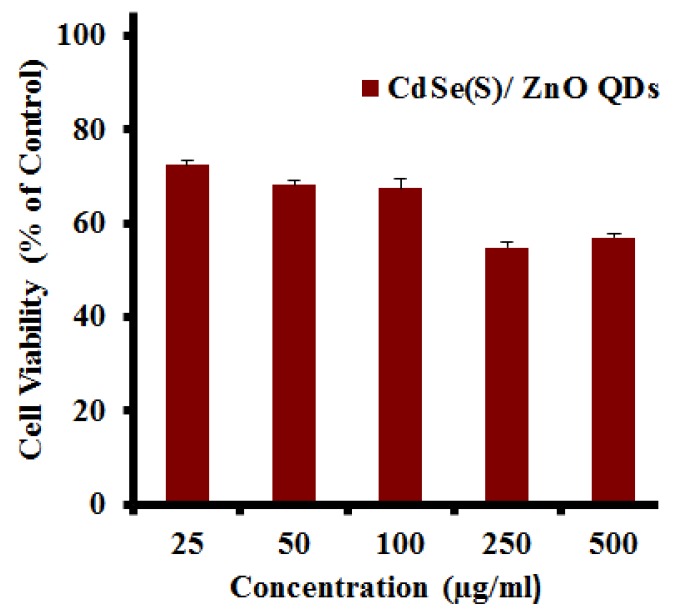
The results of cytotoxicity assays of CdSe(S)/ZnO core/shell QDs towards human colorectal carcinoma cells (HCT-116). Error bars indicate standard error of the mean and cell media without treatment with QDs was used as a control.

Core/shell QD structures, in which a relatively inert shell encapsulates the core, are widely considered to be an efficient method of attenuating the toxicity of QDs, particularly if the core contains metals such as cadmium [44,45,54,56,57,68]. The toxicity of core/shell CdSe(S)/ZnO QDs towards HCT-116 cancer cells indicated that the core/shell QDs exhibited low toxicity at all concentrations studied. As shown in Figure 7, the viability of the cells was determined to lie between 72.5 ± 1.0% and 56.9 ± 1.0% across the concentration range of 25 to 500 µg/mL, indicating that the LC_50_ of the cells is not reached even at a concentration of 500 µg/mL, the highest concentration used in this series of experiments. Clearly, the shell inhibits release of free cadmium ions, limiting cell death. However, CdSe(S)/ZnO QDs exhibit higher cytotoxicity than CdSe(S) QDs at concentrations of 25 and 50 µg/mL. The reason can be attributed to the fact that unlike CdSe(S) QDs, there is not a significant decrease in viability of HCT-116 cells towards CdSe(S)/ZnO QDs with increasing concentration. As shown in Figure 5 and Figure 7, the difference between viability of HCT-116 between concentrations of 25 and 500 µg/mL is 54% for CdSe(S) QDs and 16% for CdSe(S)/ZnO QDs, indicating that increasing concentration does not have a significant effect on the cytotoxicity of CdSe(S)/ZnO QDs. This suggests that the cytotoxicity of CdSe(S)/ZnO QDs is not related to cadmium, because the shell of zinc oxide protects the core against oxidation, but there are some un-reacted acetate ions in the aqueous solution of CdSe(S)/ZnO QDs that restrict cell viability. This is in accord with literature reports, because cytotoxic effects of sodium acetate and acetate ions on human cells have been extensively studied [77,78,79,80]. For example, it has been reported that acetate ions induce cell death and apoptosis either in colorectal carcinoma cells [77] or human gastric adenocarcinoma cell lines [78]. It has also been determined that the cytotoxicity of acetate can be induced through coupling with cellular proteins via molecular mechanisms [79] and acetate can promote the release of reactive oxygen species [80]. Therefore, reduction of cell viability of HCT-116 cells towards CdSe(S)/ZnO can be attributed to the presence of acetate ions in aqueous solution of CdSe(S)/ZnO QDs. As shown in Figure 7, cell viability decreases after incubation of the cells in the presence of CdSe(S)/ZnO QDs at concentrations ≥100 µg/mL, which is in accordance with increasing concentration of unreacted acetate ions, whilst the cytotoxicity of CdSe(S) QDs can be attributed to the presence of free cadmium ions.

#### 3.2.5. Cytotoxicity Assays towards WS1 Cells

**Figure 8:**
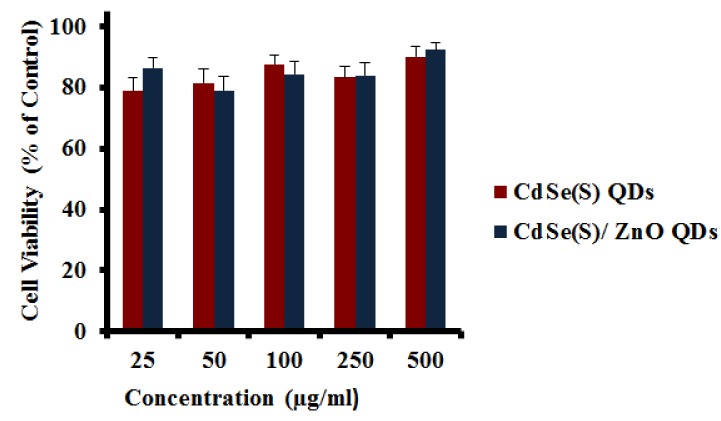
The results of cytotoxicity assays of CdSe(S) and CdSe(S)/ZnO QDs towards human skin fibroblast (WS1) cell line. Error bars indicate standard error of the mean and cell media without treatment with QDs was used as a control.

Human skin fibroblast (WS1) cells were used to determine the cytotoxicity of the as-synthesized QDs towards normal, resilient cells. The results showed that both CdSe(S) and CdSe(S)/ZnO QDs exhibited low toxicity at all of the concentrations studied (Figure 8), whilst the viability of HCT-116 cells was significantly decreased after incubation with both CdSe(S) and CdSe(S)/ZnO QDs at all concentrations (Figure 6 and Figure 7). This indicates that cell resistance is another important factor in cytotoxicity assessments.

### 3.3. Confocal Microscopy Studies

**Figure 9:**
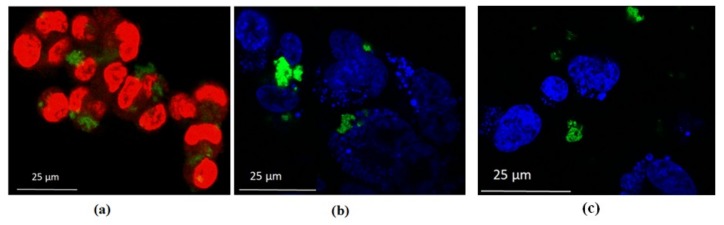
Confocal images of HCT-116 cells with QDs: (**a**) fixed cells (red) and CdSe(S) QDs (green); (**b**) live cells (blue) and CdSe(S) QDs (green); (**c**) live cells (blue) and CdSe(S)/ZnO QDs (green).

Images of HCT-116 cells after treatment with QDs confirmed the cytotoxicity data. As can be seen in Figure 9a,b, where only the cell nuclei are visible by Hoechst or DAPI staining, many cells remained stable after treatment with CdSe(S) QDs at 100 µg/mL. The images show highly luminescent CdSe(S) QDs present in both the fixed and live cells, with no determinable loss of brightness. Live HCT-116 cells were also incubated with CdSe(S)/ZnO QDs, confocal images of which showed that whilst CdSe(S)/ZnO QDs aggregated in the biological media, many cells remained stable in the presence of QDs (Figure 9c). The uptake mechanism is unknown, but as shown in the confocal images (Figure 9), QDs tended to adhere to the nuclei rather than entering within the cell nucleus, presumably due to inadequate interactions between the QDs and cell nuclei, thereby prohibiting uptake.

**Figure 10:**
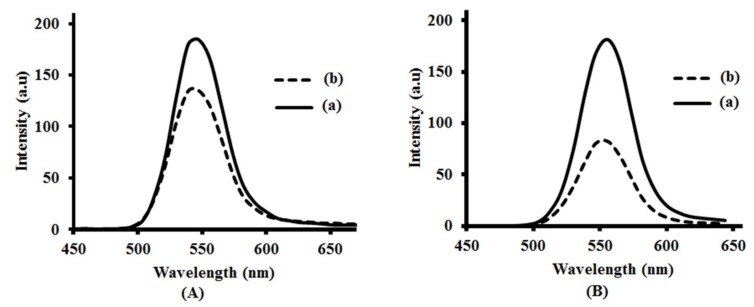
Photoluminescence spectra of CdSe(S) QDs (**A**) and CdSe(S)/ZnO QDs (**B**): (**a**) in water and (**b**) in cell media. The excitation wavelength of the instrument was adjusted at 405 nm at the time of measuring the photoluminescence (PL).

The emission profiles of QDs in both the cell media and water were recorded using a confocal microscope in order to determine any changes in photoluminescence spectra of QDs in cell media (Figure 10A). The images of QDs in cell media were also recorded and described in Supplementary Materials Data S8, indicating that QDs are stable in cell media. The photoluminescence emission of CdSe(S) QDs were found with no change in cell media, in contrast to literature reports, which have indicated that water soluble MPA-capped CdTe QDs had an altered emission profile in cell growth media [81]. Moreover, we have previously shown that the optical properties of CdSe(S) QDs after binding to antibodies remain unchanged [82]. Emission spectra indicated that CdSe(S) QDs have a maximum emission at 548 nm in the cell media, with no significant change or shift in photoluminescence relative to that in water. The emission of CdSe(S)/ZnO QDs was found to have a lower intensity and shift to 546 nm in cell media from 556 nm in water (Figure 10B).

## 4. Conclusions

We have shown that the cytotoxicity of QDs can be controlled with the aqueous synthesis of stable QDs. It was determined that coating of QDs with a ZnO shell protects the core against oxidation and the production of toxic free radicals, resulting in decreased cytotoxicity of QDs, even at high concentrations. Images of cells after incubation with QDs at a concentration of 100 µg/mL indicated that the cells remained viable, confirming the cytotoxicity data. The stability of the QD cores in the cell media was found to be related to the overall toxicity of QDs, which is largely governed by the free-ion concentrations.

## Figures and Tables

**Table 1:** Elemental analysis of QDs.

QD Type	Elemental %					
	Carbon	Oxygen	Selenium	Cadmium	Sulfur	Zinc
CdSe(S)	42.5	29.9	0.7	13.7	13.1	0
CdSe(S)/ZnO	20.5	37.7	0.6	16.8	12.3	12

## Supplementary Materials

The following are available online at https://www.mdpi.com/2079-4991/9/3/465/s1.
