# Supplementary material for: Nanoparticles for Bioapplications: Study of the Cytotoxicity of Water Dispersible CdSe(S) and CdSe(S)/ZnO Quantum Dots
Source: Nanomaterials (Basel). 2019 Mar 20;9(3):465. doi: 10.3390/nano9030465 (PMC6474084; doi:10.3390/nano9030465)
Supplement: Supplementary file 1 [file nanomaterials-09-00465-s001.pdf]

Supplementary Information

Nanoparticles for Bioapplications: Study of the Cytotoxicity of Water Dispersible CdSe(S) and CdSe(S)/ZnO Quantum Dots

Fatemeh Mirnajafizadeh [1,†], Deborah Ramsey [1,†], Shelli McAlpine [1], Fan Wang [2] and John Arron Stride [1,*]

[1] School of Chemistry, University of New South Wales, Sydney, NSW 2052, Australia; f.mirnajafizadeh@unsw.edu.au (F.M.); d.ramsey@unsw.edu.au (D.R.); s.mcalpine@unsw.edu.au (S.M.)
[2] School of Mathematical and Physical Sciences, University of Technology Sydney, Ultimo, Sydney, NSW 2007, Australia; Fan.Wang@uts.edu.au
* Correspondence: j.stride@unsw.edu.au
† These authors contributed equally in this work.

S1. Synthesis of Water Dispersible QDs

S.1.1. Synthesis of CdSe(S) QDs: CdSe(S) QDs were prepared according to a modified literature method [1]. Briefly, a Cd-MPA precursor solution was prepared by dissolving 1.48 g (6.5 mmol) $CdCl_2$ in 100 mL ultrapure water and adding MPA (0.7 mL) to the solution making the molar ratio of Cd:MPA 6.5:8; the pH was adjusted to 12.5 using 1M NaOH, this was labelled Sample 1. Sample 1 was then placed under nitrogen for 10 minutes and NaHSe (1 mmol, 20 mL), was injected to the solution to form a Cd-Se-MPA complex as a clear yellow liquid; this was labelled as Sample 2. Sample 2 was transferred to a Teflon-lined autoclave and placed in a conventional oven for 1 h, under hydrothermal treatment at T = 150° C. Finally, CdSe-MPA capped QDs were formed in an aqueous orange solution and labelled as Sample 3. The as-prepared QDs were orange in colour in contrast to that previously reported [1], with a yellow emission colour under UV light.

S.1.2. Synthesis of CdSe(S)/ZnO QDs: CdSe(S) QDs were prepared, as described in section S.1.1 and coated with ZnO according to previous literature report [1], based upon the controlled hydrolysis of zinc salts at basic pH. First, an aqueous suspension of as-prepared CdSe(S) QDs (Sample 3, described in S.1.1), was diluted using ultrapure water. The concentration of CdSe(S) QDs was 1.7 mg in 5 mL and the pH was adjusted to 13 using 1M NaOH. This solution was refluxed for 2 h before injecting an aqueous solution of $Zn(OAC)_2 \cdot 2H_2O$ (18 mL, 0.01 M) at T = 100 °C. Reflux was continued for another hour and CdSe(S)/ZnO core/shell QDs were obtained as orange nanoparticles.

S2. Cell Culture of HCT-116 and WS1 Cell Lines

Cell cultures of human colorectal carcinoma cells (HCT-116) and human skin fibroblast cell line (WS1) were obtained according to the standard protocol [2]. As cells are usually received frozen in the culture medium with 5–10% dimethyl sulfoxide (DMSO) in the vapour of liquid nitrogen at 77 K, they were first defrosted to 37 °C and the culture medium discarded by using a centrifuge at 1500 rpm for 5 min. Then, the HCT-116 and WS1 cells were separately resuspended in Dulbecco's Modified Eagle Medium (DMEM) supplemented with 10% fetal bovine serum (FBS), 1% penicillin/streptomycin, (1%) nonessential amino acid and L-glutamine (2 mM) as growth medium and transferred in two 75 cm^2 flasks. Finally, the cells were grown in a humidified incubator at 37 °C with 5% CO_2 and as cells reached confluence; they were immediately used for cytotoxicity tests in separate experiments.

S3. Preparation of HCT-116 Cells-CdSe(S) QDs Samples

S.3.1. Fixed HCT 116 cells-CdSe(S) QDs sample: cells were cultured (as described in S2) and were seeded in 35 mm fluorodish cell culture dishes at a density of 20000 cell/dish for 24 h. The cells were then treated with an aqueous solution of CdSe(S) QDs (250 μg/mL) and incubated at 37 °C with 5% CO_2 for 24 h. After incubation, the cells were fixed with 4% paraformaldehyde in PBS for 1 hour, before finally being washed with PBS and stained with 5 μg/mL bisbenzimide (Hoechst 33342) for 20 min in room temperature.

S.3.2. Live HCT 116 cells-QDs samples: cells were cultured according to the standard protocol [2] similar to the fixed cells and were seeded in a 24-well glass bottom plate at a density of 20000 cells/dish for 24 h. Then, cells were treated with an aqueous solution of CdSe(S) QDs (100 μg/mL) and incubated at 37 °C with 5% CO_2 for 24 h. As-prepared samples were used for confocal microscopy studies.

S4. The Comparison of PXRD of CdSe(S) QDs with Standard Peaks

The diffraction peaks of CdSe(S) QDs were observed between standard cubic CdSe [3] and CdS [4], as shown in Table S1.

Table S1. The comparison of diffraction peaks of as-synthesized CdSe(S) QDs with cubic CdSe and CdS standard peaks.

h k l	CdSe cubic (Standard pattern)	As –synthesized CdSe(S) QDs	CdS cubic (Standard pattern)
1 1 1	25.35	26.29	26.50
2 2 0	42.00	43.66	43.96
3 1 1	49.69	51.67	52.13

S5. Dynamic Light Scattering of CdSe(S) QDs

The particle size distribution was determined using DLS and the obtained size distribution histograms showed that the QDs are well dispersed in water. The average size of MPA-capped CdSe(S) QDs in aqueous solution is about 7.16 nm with narrow size distribution. Figure S1 shows a typical histogram of as-prepared QDs. The hydrodynamic diameter is about two times larger than the estimated size with HRTEM and PXRD due to the fact that the hydrodynamic diameter contains organic molecules on the surface of QDs, originating from capping agent (MPA), whereas HRTEM, PXRD reflect the particle size of the inorganic core of the nanoparticles in accord with literature [5].

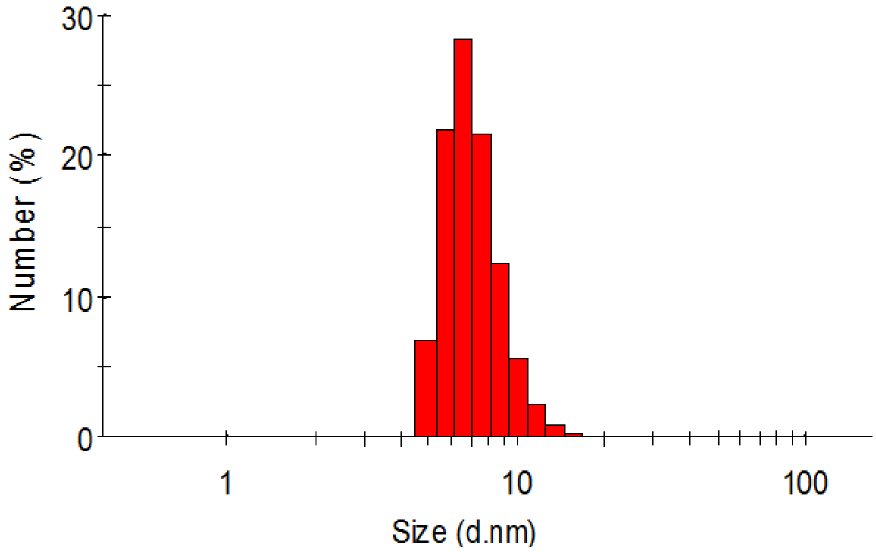

Figure S1. Size distribution histogram of CdSe(S) QDs.

S6. Determination of Quantum Yield (QY) of QDs

The quantum yield (QY) of as-synthesized CdSe(S) and CdSe(S)/ZnO QDs was determined using a method described in literature [6,7]. This method is based upon comparison of the QY of a known standard fluorescent dye with QY of as-synthesized QDs using following equation:

$$\phi x = \phi\, ST\left(\frac{Grad\ X}{Grad\ ST}\right)\left(\frac{\eta 2\ X}{\eta 2\ ST}\right) \quad (1)$$

where subscripts X and ST represent the sample and standard dye, ϕ is the QY, η is the refractive index of the solvent and *Grad* is gradient of the graphs of integrated fluorescence intensity vs absorbance.

To determine the QY of the obtained QDs, Rhodamine 6G was used as a standard dye due to having an excitation profile at 526 nm and an emission at 555 nm along with yellow emitting, which is comparable with both CdSe(S) and CdSe(S)/ZnO QDs, as shown in Figure S2. The QY of Rhodamine 6 G is 95% in water ($\lambda_{excitation}$ = 248–528 nm), as reported in literature [8].

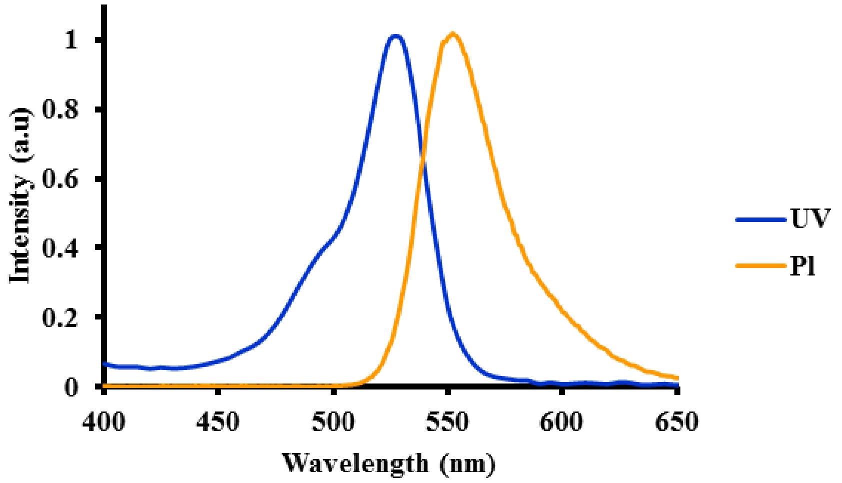

Figure S2. Optical properties of Rhodamine 6 G: (blue) UV & (yellow) PL.

First, the emission and absorption of Rhodamine 6G and QDs were recorded in a range of diluted samples. The pathlength of cuvette was 10 mm, the absorption was recorded up to a value of 0.1. Ultra-pure water was used as the solvent in all measurements. Then, the integrated fluorescence intensity was calculated using Origin software for each sample and linear graphs were plotted according to the values of the integrated intensity and recorded absorbance for all samples, as shown in Figure S3.

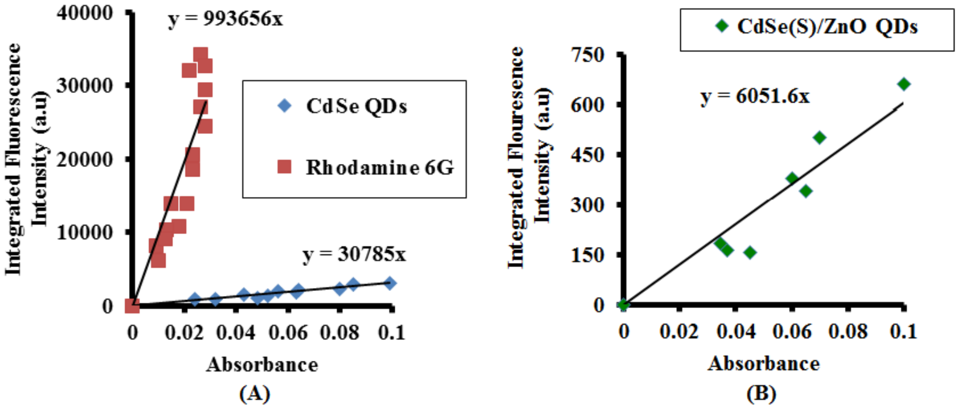

Figure S3. (A) Linear plots of Rhodamine 6G (red) & CdSe(S) QDs (blue) and. (B) CdSe(S)/ZnO QDs.

Finally, the Equation 1 was used, as below:

$\phi x = \phi\, ST (\frac{Grad\ X}{Grad\ ST})(\frac{\eta 2\ X}{\eta 2\ ST})$ ($\phi\, ST$ = QY of Rhodamine 6G = 95%)

QY for CdSe(S) QDs: $\phi x = 0.95\ (\frac{30785}{993656})$ = 0.0294 ~ 3%

QY for CdSe(S)/ZnO QDs: $\phi x = 0.95\ (\frac{6051.6}{993656})$ = 0.00578 ~ 0.6%

S7. Images of QDs under UV Light

CdSe(S) and CdSe(S)/ZnO QDs were found to have $\lambda_{emission}$ = 550 nm and 560 nm, corresponding to the green and yellow coloured samples, respectively (Figure S4).

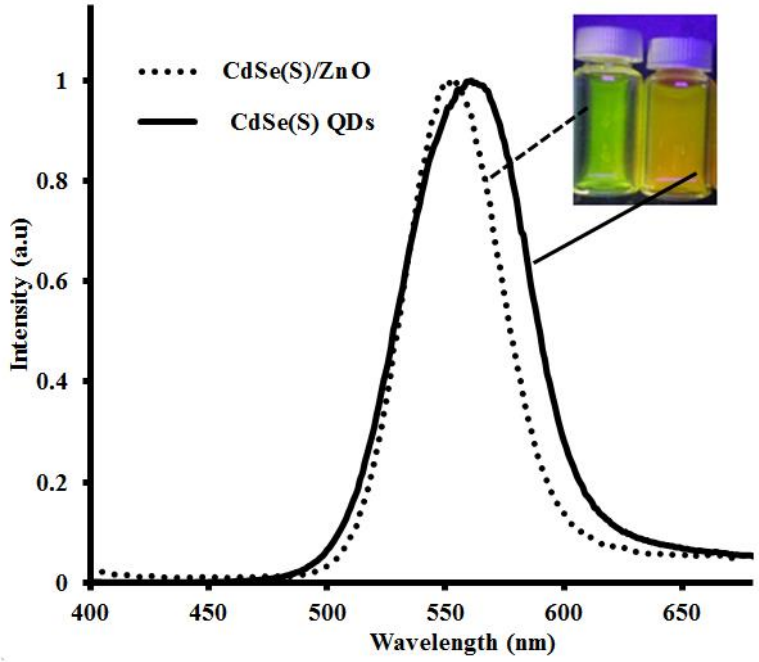

Figure S4. Photoluminescence spectra of QDs ($\lambda_{excitation}$ = 350 nm): (a) CdSe(S) QDs, (b) CdSe(S)/ZnO QDs and (inset) the images of QDs under UV light ($\lambda_{excitation}$ = 365 nm): CdSe(S) QDs (yellow) and CdSe(S)/ZnO QDs (green).

S8. Confocal Images of QDs in Cell Media

Confocal images of CdSe(S) and CdSe(S)/ZnO QDs were recorded using Zeiss LSM 780 confocal microscopy and showed that QDs are stable in both cell media and water, as shown in Figure S5.

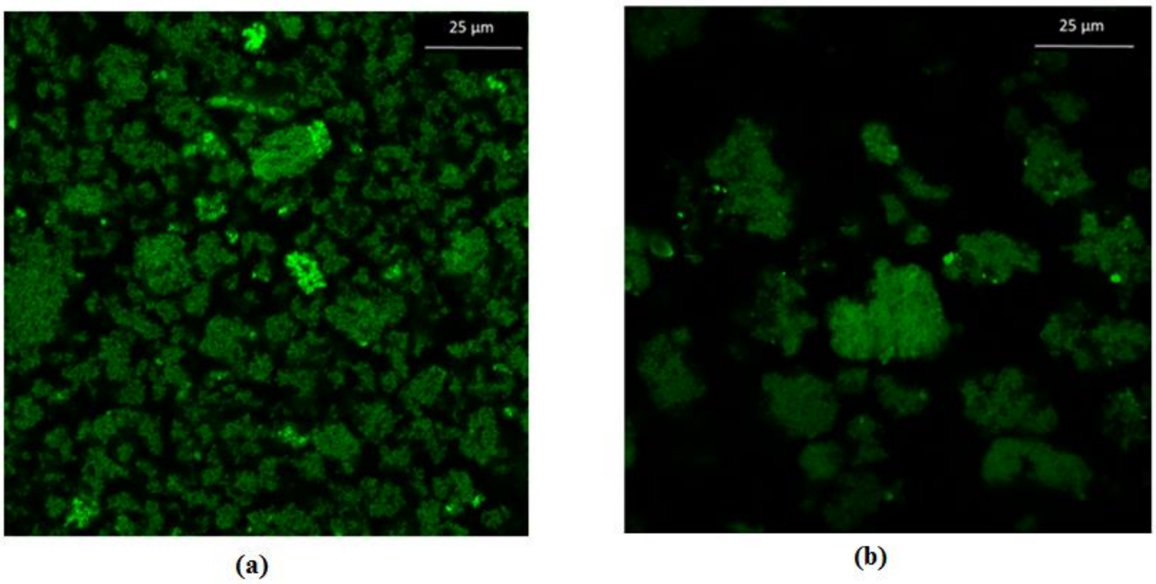

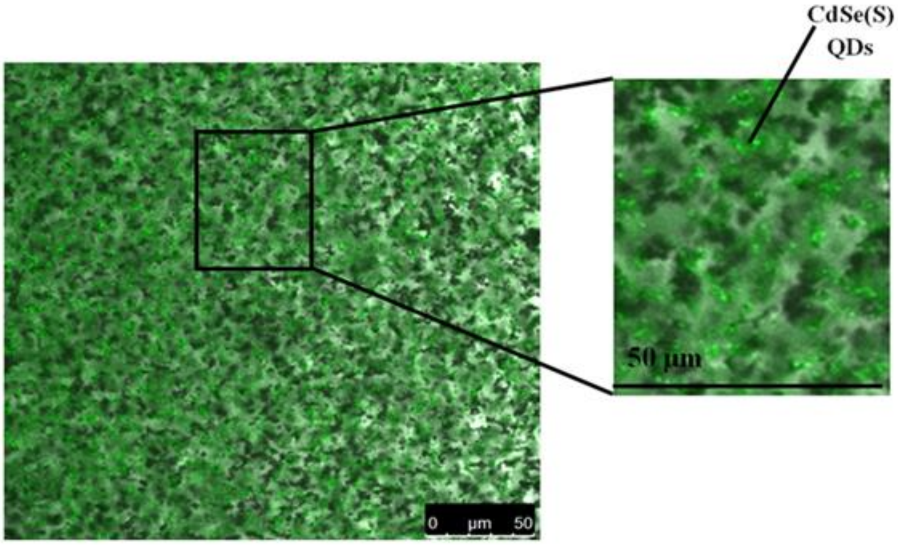

(c)

Figure S5. Confocal image of as-prepared QDs: QDs can be observed as green illuminated small dots: (a) CdSe(S) QDs in cell media, (b) CdSe(S)/ZnO QDs in cell media and (c) CdSe(S) QDs in water.

References

[1]. F. Aldeek, C. Mustin, L. Balan, G. Medjahdi, T. Roques-Carmes, P. Arnoux, R. Schneider, Enhanced Photostability from CdSe(S)/ZnO Core/Shell Quantum Dots and their use in Biolabeling, *Eur. J. Inorg. Chem.*, (2011) 794-801.

[2]. M. C. Phelan, In Current Protocols in Cell Biology, John Wiley & Sons, Inc., 2007, pp. 1.1.1-1.1.18.

[3]. The International Centre for Diffraction Data (ICDD), 1978, JCPDs files, No. 00.19-0191.

[4]. The International Centre for Diffraction Data (ICDD), 1978, JCPDs files, No. 00-010-0454.

[5]. J. Wang, H. Han, Hydrothermal synthesis of high-quality type-II CdTe/CdSe quantum dots with near-infrared fluorescence. *J. Colloid Interface Sci.*, 351 (2011) 83-87.

[6] Y.A. Wu, J.H. Warner, Shape and property control of Mn doped ZnSe quantum dots: from branched to spherical, *J. Mater. Chem.*, 22 (2012) 417-42.

[7]. F. Mirnajafizadeh, D. Ramsey, S. McAlpine, F. Wang, P. Reece, J.A. Stride, Hydrothermal synthesis of highly luminescent blue-emitting ZnSe(S) quantum dots exhibiting low toxicity, *Mater. Sci. Eng., C*, 64 (2016) 167-172.

[8]. R. F. Kubin, N. Feletcher, fluorescence quantum yields of some Rhodamine dyes, *J. Luminescence*, 27 (1982) 455-462.
